# Germline Variants and Genetic Interactions of Several EMT Regulatory Genes Increase the Risk of HBV-Related Hepatocellular Carcinoma

**DOI:** 10.3389/fonc.2021.564477

**Published:** 2021-06-11

**Authors:** Wen-Xuan Liu, Lei Yang, Hui-Min Yan, Li-Na Yan, Xiao-Lin Zhang, Ning Ma, Long-Mei Tang, Xia Gao, Dian-Wu Liu

**Affiliations:** ^1^ Department of Epidemiology and Statistics & Hebei Province Key Laboratory of Environment and Human Health, School of Public Health, Hebei Medical University, Shijiazhuang, China; ^2^ Department of Laboratory Medicine, Shijiazhuang Fifth Hospital, Shijiazhuang, China; ^3^ Department of Social Medicine and Health Care Management & Hebei Province Key Laboratory of Environment and Human Health, School of Public Health, Hebei Medical University, Shijiazhuang, China

**Keywords:** hepatocellular carcinoma, epithelial-mesenchymal transition, polymorphisms, interaction, multifactor dimensionality reduction

## Abstract

Epithelial-mesenchymal transition (EMT) plays an important role in the development of hepatitis B virus (HBV)-related hepatocellular carcinoma (HCC). We hypothesized that germline variants in the major EMT regulatory genes (*SNAIL1*, *ZEB1*, *ZEB2*, *TWIST1*) may influence the development of HBV-related HCC. We included 421 cases of HBsAg-positive patients with HCC, 1371 cases of HBsAg-positive subjects without HCC [patients with chronic hepatitis B (CHB) or liver cirrhosis (LC)] and 618 cases of healthy controls in the case-control study. Genotype, allele, and haplotype associations in the major EMT regulatory genes were tested. Environment-gene and gene-gene interactions were analysed using the non-parametric model-free multifactor dimensionality reduction (MDR) method. The *SNAIL1*rs4647958T>C was associated with a significantly increased risk of both HCC (CT+CC *vs*. TT: *OR*=1.559; 95% confidence interval [*CI*], 1.073-2.264; *P*=0.020) and CHB+LC (CT+CC *vs*. TT: *OR*=1.509; 95% *CI*, 1.145-1.988; *P*=0.003). Carriers of the *TWIST1*rs2285681G>C (genotypes CT+CC) had an increased risk of HCC (CG+CC *vs*. GG: *OR*=1.407; 95% *CI*, 1.065-1.858; *P*=0.016). The *ZEB2*rs3806475T>C was associated with significantly increased risk of both HCC (*P*_recessive_ =0.001) and CHB+LC (*P*_recessive_<0.001). The CG haplotype of the rs4647958/rs1543442 haplotype block was associated with significant differences between healthy subjects and HCC patients (*P*=0.0347). Meanwhile, the CT haplotype of the rs2285681/rs2285682 haplotype block was associated with significant differences between CHB+LC and HCC patients (*P*=0.0123). In MDR analysis, the combination of *TWIST1*rs2285681, *ZEB2*rs3806475, *SNAIL1*rs4647958 exhibited the most significant association with CHB+LC and Health control in the three-locus model. Our results suggest significant single-gene associations and environment-gene/gene-gene interactions of EMT-related genes with HBV-related HCC.

## Highlights

1. The functional *SNAIL1* exon variant rs4647958T>C, the *ZEB2* promoter exon variant rs3806475T>C and the *TWIST1* promoter exon variant rs2285681G>C are associated with increased risk of HBV-related HCC.
2. The CG haplotype of the rs4647958/rs1543442 haplotype block was associated with significant differences between healthy control subjects and HCC patients. Additionally, the CT haplotype of the rs2285681/rs2285682 haplotype block was associated with significant differences between CHB+LC and HCC patients.
3. *TWIST1* rs2285681 and *SNAIL1* rs4647958 showed a significant environment-gene interaction for the development of HCC.

## Introduction

Hepatocellular carcinoma (HCC), a common malignant tumour of the digestive system, is the second leading cause of cancer-related death in China. HCC is characterized by high malignant potential, concealed pathogenesis, rapid progress, poor prognosis and a high mortality rate. It is typically diagnosed during the middle and late disease stages, when surgery is no longer a viable option (1). Therefore, it is important to identify genetic loci that may be valuable predictors for the development of both HCC and chronic hepatitis B virus (HBV) infection in order to evaluate the risk of HCC in patients with HBV infection.

In recent years, the significance of epithelial-mesenchymal transition (EMT) in tumours has been extensively studied. There are many complex factors that may influence the process of tumour metastasis; however, the specific underlying mechanisms are not yet clear. A great many studies have revealed that EMT plays an important role in tumour invasion and metastasis. To date, three well-established transcriptional regulatory groups have been identified as important factors in regulating the expression of EMT molecular markers (2). Studies have shown that several EMT regulators are involved in the process of tumour metastasis and that the phenotypic changes associated with EMT play a key role in the development of invasive phenotypes in colon cancer, thyroid cancer and breast cancer (3). In addition, increasing evidence demonstrates that EMT is involved in promoting other aspects of tumour progression (4–6). A more comprehensive understanding of the role of EMT in regulating the growth and metastasis of tumours is critical for improving the diagnosis and treatment of these tumours.

Previous work has demonstrated that *SNAIL* and *TWIST* are the major regulators of EMT, which subsequently induces HCC (7). Overexpression of *SNAIL* and *TWIST* is associated with greater tumour volume, increased recurrence, and shorter disease-free and overall survival in HCC patients (7). In addition, *SNAIL* and *TWIST* expression is associated with decreased E-cadherin expression in HCC. *In vitro* experiments have confirmed that overexpression of *SNAIL* or *TWIST* promotes invasion and increases the interstitial phenotype of tumour cells. Overexpression of *SNAIL* or *TWIST* in Huh7 cells suppresses E-cadherin expression and induces EMT (3, 8, 9). In addition, previous studies have demonstrated that EMT leads to increased chemotherapeutic resistance in poorly differentiated HCC cell lines (4–6). Wu et al. constructed gemcitabine-resistant HCC cell lines and found that these cells develop an EMT-related phenotype (10). Furthermore, real-time PCR has been used to demonstrate the downregulation of E-cadherin expression and increased expression of *TWIST*1, further confirming the development of EMT (11).

Genetic variations in EMT-related regulatory genes may affect the process of EMT and thus influence the development of HCC or chronic HBV infection. However, there has been no published research on the association of these variants with HCC or chronic HBV infection risk. Moreover, although several genetic variants associated with these liver diseases have been revealed by GWAS, little research has been done on the link between these genes and disease progression. Therefore, it is of great value to identify which genetic loci of EMT-related genes are related with the development of HCC. Thus, we assessed whether Genetic variations in EMT-related regulatory genes are associated with the progress of HCC and chronic HBV infection.

A common analysis method for genotype data is to perform a single gene locus or haplotype analysis on a single gene, that is, to detect the association between each locus or gene and disease separately. However, when we want to explain the genetic changes in complex diseases, the usefulness of this analysis is limited (12). Because the risk of a particular disease may be explained by genetic mutations at other loci, discovering gene-to-gene interactions is more conducive to a comprehensive understanding of the factors that affect disease risk (13). In this study, we investigated possible genetic interactions between EMT-related genes (*SNAIL1, ZEB1, ZEB2* and *TWIST1*) in HBV-related HCC in the Han population and their relevance as potential biomarkers for HBV and HCC. This approach may help develop new therapy or individualized treatments for HBV-related HCC and chronic HBV infection.

## Materials and Methods

### Study Subjects

Case-control studies were conducted to investigate HBV-related HCC and chronic HBV infection in northern China. To evaluate HBV-associated mutations and their correlation with HCC risk, 421 HBsAg-positive patients with HCC, 1371 HBsAg-positive patients without HCC [691 cases of chronic hepatitis B (CHB) and 680 cases of liver cirrhosis (LC)] and 618 controls without HBV infection were enrolled. All subjects are independent of each other and are ethnically Han Chinese. All participants were recruited between January 2010 and March 2014 from the First, Second and Fourth Hospitals of Hebei Medical University and the Fifth Hospital of Shijiazhuang City. Each subject provided demographic characteristics as well as a one-time 2 mL blood sample. All subjects signed a written informed consent forms to study initiation. This study was approved by the institutional review board of Hebei Medical University (Ethics Committee of Hebei Medical University: No. 2017053).

Healthy individuals were defined as (i) HBsAg, antibodies against HBc (anti-HBc) and other HBV biomarkers were free; (ii) blood routine and biochemical indexes were normal; (iii) without a history of hepatitis B vaccination; (iv) without endocrine, cardiovascular, renal or other liver diseases. CHB patients were defined as (i) serum HBsAg was positive; (ii) HBeAg was positive; (iii) anti-HBe was negative; (iv)serum HBV-DNA >2000 IU/mL lasting for >6 months; (v) the value of alanine aminotransferase (ALT) was persistent or repeated rising; (vi) liver histology showed hepatitis. LC patients were defined by clinical manifestations of portal hypertension (e.g., varicose oesophageal or gastric fundus, ascites and splenomegaly) and imaging results of ultrasonography, computed tomography, and magnetic resonance imaging (14, 15). HBV-related HCC patients were defined as pathologic diagnosis and/or blood alpha-fetoprotein (AFP) >400 ng/mL, at the same time combined with imaging examination results (16, 17). Patients were excluded from this study if they with alcoholic liver disease, positive laboratory tests for HCV (identified by the presence of anti-HCV and/or HCV-RNA) and HIV or suspected autoimmune diseases with an antinuclear antibody titre greater than 1:160.

The personal information of the research subjects was obtained through questionnaires, which included the subjects’ gender, age, smoking status, and drinking status. The definition of smoking and drinking here is: an individual who smokes every day and has smoked for more than 1 year is defined as a smoker, and an individual who drinks once or more a week for more than 6 months is defined as a drinker. We collected about 2 mL of anticoagulated venous blood by ethylenediamine tetra-acetic acid (EDTA) from each subject. Each subject signed an informed consent form. The study protocol adhered to the ethical guidelines set forth by the 1975 Declaration of Helsinki and was approved by the Hebei Medical University ethics committee.

### Polymorphisms Selection and Genotyping

According to the dbSNP database (http://www.ncbi.nlm.nih.gov/), we selected 6 EMT gene loci located in the promoter, regulator coding region and 3’-UTR. All putative functional single-nucleotide polymorphisms (SNPs) of the genes encoding the aforementioned EMT regulators (*SNAIL1* rs4647958T>C, *SNAIL1* rs1543442G>A, *ZEB1* rs7349C>T, *ZEB2* rs3806475T>C, *TWIST1* rs2285681G>C and *TWIST1* rs2285682T>G) with a minor allele frequency greater than 5% in the Chinese population were selected. The location information in gene region for the selected SNPs was shown in **Table 1**. A Genomic DNA Purification Kit purchased from Promega was used for genomic DNA extraction and time of flight mass spectrometry technology from SOLARBIO Technology Co., Ltd. was used for all sample SNP genotyping. Primers for the five SNP alleles were designed by the Bio Miao Biological Company with the aid of MassARRAY^®^ Assay Design 4.0 Software (Sequenom Inc., San Diego, CA, USA). SNPs were genotyped using TaqMan-based PCR. Basic information for the selected SNPs was shown in **Supplementary Table 1**.

**Table 1:** Associations between the SNPs in candidate EMT regulators and risk of chronic HBV infection in the discovery set.

SNP	Location in Gene Region	HCC*n* = 421	CHB+LC*n* =1371	Health Control*n* = 618	MAF			*P* ^a^	*P* ^b^	*P* ^c^
					HCC	CHB+LC	Health Control			
rs4647958T>C	*SNAIL1*, exon	338/78/2	1106/246/8	530/70/5	0.098	0.096	0.066	0.009	0.728	0.001
rs1543442G>A	*SNAIL1*, 3’-UTR	168/194/57	544/621/196	226/293/95	0.368	0.372	0.393	0.715	0.694	0.421
rs7349C>T	*ZEB1*, 3’-UTR	269/135/14	848/452/54	409/173/29	0.195	0.207	0.189	0.485	0.920	0.068
rs3806475T>C	*ZEB2*, promoter	120/216/84	305/769/287	133/237/72	0.457	0.493	0.431	<0.001	0.020	<0.001
rs2285681G>C	*TWIST1*, promoter	209/173/33	704/541/95	343/223/48	0.288	0.273	0.260	0.043	0.516	0.180
rs2285682T>G	*TWIST1*, promoter	331/80/8	1026/297/35	481/119/11	0.115	0.135	0.115	0.800	0.247	0.228

^a^HCC vs health control; ^b^HCC vs CHB+LC; ^c^CHB+LC vs health control.

P value of association test from the best-fitted genetic model calculated by the unconditional logistic regression, adjusted for age, gender, smoked and drink, which owned the smallest Akaikein formation criterion value.

### Statistical Analysis

SPSS version 18.0 (SPSS Inc., Chicago, IL, USA), Haploview 4.2 software (Copyright (c) 2003-2006 Broad Institute of MIT and Harvard, United States) and MDR 3.0.2 software (https://sourceforge.net/projects/mdr/) were used to perform statistical analyses. Categorical variables were described using frequencies, while continuous data with abnormal distribution were described using the median and interquartile range. The comparisons of continuous data sets were done using Kruskal-Wallis *H* test and evaluation of differences in categorical variables between groups was done using Pearson chi-square test. The bonfferny method was used for pairwise comparisons between groups when there was a significant difference in the overall distribution of each factor in the three groups. Calculation of odds ratios (*OR*) and 95% confidence intervals (95% *CI*) was done using unconditional logistic regression. Analysis of correlations between genetic variants and HCC stages was done by Spearman’s rank correlation. Linkage disequilibrium (LD) and haplotype block analyses were used to investigate the LD of EMT SNPs using Haploview 4.2 software. Multifactor dimensionality reduction (MDR) method as a nonparametric alternative was used to analyse the environment-gene and gene-gene interactions. The MDR analyses were performed by MDR 3.0.2 software. This extensive search for genetic interactions was done for HCC. Up to four loci interactions were tested using 10-fold crossvalidation in a search considering all possible SNP combinations. The SNP combination with maximum cross-validation consistency (CVC) was considered to be the best model (*see* METHODS in the **Supplements**). All hypothesis tests were based on two-sided. When *P* values were less than 0.05, it is considered statistically significant.

## Results

### Subject Characteristics

Baseline characteristics of the 1371 HbsAg-positive patients without HCC (CHB+LC), 421 HBsAg-positive patients with HCC and 618 healthy control subjects were shown in **Supplementary Table 2**. The age, gender, and tobacco and alcohol use distributions were significantly different among all studied groups (*P*<0.05). Smoking and drinking were significantly lower in healthy patients versus in HBsAg-positive patients with and without HCC. The proportion of males was higher in the HBsAg-positive patients versus the healthy subjects, while patients older than 45 years old were more frequent in the HBsAg-positive patients with HCC. We adjusted for these factors in the multivariate logistic regression models.

### Genotypes of EMT Regulators and Their Association With Hepatocellular Carcinoma and Chronic HBV Infection Risk

The genotype distributions of the six EMT regulators and their associations with HCC and CHB+LC are presented in **Tables 1** and **2**. Based on the best genetic model (defined as the model with the smallest AIC value), the *SNAIL1* exon variant rs4647958T>C was significantly associated with an increased risk of both HCC (*P*_dominant_ =0.020) and CHB+LC (*P*_dominant_ =0.003). The *ZEB2* promoter variant rs3806475T>C was significantly associated with an increased risk of both HCC (*P*_recessive_ =0.001) and CHB+LC (*P*_recessive_<0.001). Further, the *TWIST1* promoter variant rs2285681G>C was significantly associated with an increased risk of HCC (*P*_dominant_ =0.016). However, no significant association was observed between any of the other loci and the risk of HbsAg-positive HBV with or without HCC. Therefore, we further analysed the *SNAIL1* rs4647958T>C, *ZEB2* rs3806475T>C and *TWIST1* rs2285681G>C SNPs. As shown in **Table 2**, the rs4647958T>C SNP was associated with a significantly increased risk of both HCC (CT+CC *vs*. TT: *OR*=1.559; 95% confidence interval [*CI*], 1.073-2.264; *P* = 0.020) and CHB+LC (CT+CC *vs*. TT: *OR*=1.509; 95% confidence interval [*CI*], 1.145-1.988; *P* = 0.003) under the dominant model. Carriers of the *TWIST1* rs2285681G>C genotypes (CT+CC) had an increased risk of HCC (CG+CC *vs*. GG: *OR*=1.407; 95% confidence interval [*CI*], 1.065-1.858; *P* = 0.016) under the dominant model.

**Table 2:** Associations between the SNPs in EMT regulators and diseases risk under different genetic models.

SNP	Gene	Additive model		Dominant model		Recessive model	
		*OR*	*P* value	*OR*	*P* value	*OR*	*P* value
*HCC vs. Health*							
rs4647958T>C	*SNAIL1*	0.343(0.068-1.728)	0.194	1.559(1.073-2.264)	0.020	0.316(0.063-1.593)	0.163
rs1543442G>A	*SNAIL1*	0.841(0.552-1.282)	0.420	0940(0.711-1.245)	0.668	0.853(0.578-1.259)	0.424
rs7349C>T	*ZEB1*	0.733(0.360-1.492)	0.391	1.063(0.797-1.418)	0.679	0.707(0.350-1.430)	0.335
rs3806475T>C	*ZEB2*	1.327(0.853-2.065)	0.210	0.701(0.512-0.961)	0.027	1.918(1.313-2.803)	0.001
rs2285681G>C	*TWIST1*	1.201(0.712-2.025)	0.492	1.407(1.065-1.858)	0.016	1.023(0.617-1.697)	0.928
rs2285682T>G	*TWIST1*	1.263(0.451-3.537)	0.657	1.112(0.793-1.559)	0.538	1.240(0.444-3.464)	0.682
*HCC vs. CHB+LC*							
rs4647958T>C	*SNAIL1*	0.899(0.162-4.982)	0.903	1.122(0.831-1.516)	0.452	0.881(0.159-4.872)	0.884
rs1543442G>A	*SNAIL1*	0.969(0.673-1.394)	0.864	1.065(0.839-1.352)	0.605	0.923(0.658-1.295)	0.642
rs7349C>T	*ZEB1*	0.895(0.470-1.707)	0.737	0.958(0.751-1.223)	0.732	0.906(0.478-1.718)	0.763
rs3806475T>C	*ZEB2*	0.750(0.531-1.059)	0.102	0.692(0.530-0.904)	0.007	0.984(0.736-1.316)	0.916
rs2285681G>C	*TWIST1*	1.091(0.694-1.714)	0.706	1.145(0.904-1.450)	0.262	1.024(0.661-1.587)	0.916
rs2285682T>G	*TWIST1*	0.667(0.297-1.499)	0.327	0.792(0.598-1.051)	0.106	0.699(0.312-1.567)	0.385
*CHB+LC vs. Health*							
rs4647958T>C	*SNAIL1*	0.448(0.166-1.209)	0.113	1.509(1.145-1.988)	0.003	0.416(0.154-1.122)	0.083
rs1543442G>A	*SNAIL1*	0.860(0.642-1.152)	0.311	0.876(0.719-1.069)	0.193	0.921(0.705-1.204)	0.548
rs7349C>T	*ZEB1*	0.885(0.553-1.418)	0.612	1.211(0.988-1.484)	0.065	0.820(0.515-1.307)	0.404
rs3806475T>C	*ZEB2*	1.745(1.253-2.430)	0.001	0.975(0.772-1.230)	0.829	1.988(1.503-2.630)	<0.001
rs2285681G>C	*TWIST1*	0.958(0.659-1.393)	0.824	1.157(0.953-1.404)	0.141	0.889(0.617-1.280)	0.528
rs2285682T>G	*TWIST1*	1.469(0.736-2.931)	0.274	1.213(0.961-1.530)	0.104	1.415(0.710-2.818)	0.324

OR, odds ratio; adjusted in a logistic regression model that included age, gender, smoking and drinking.

**Figure 1:**
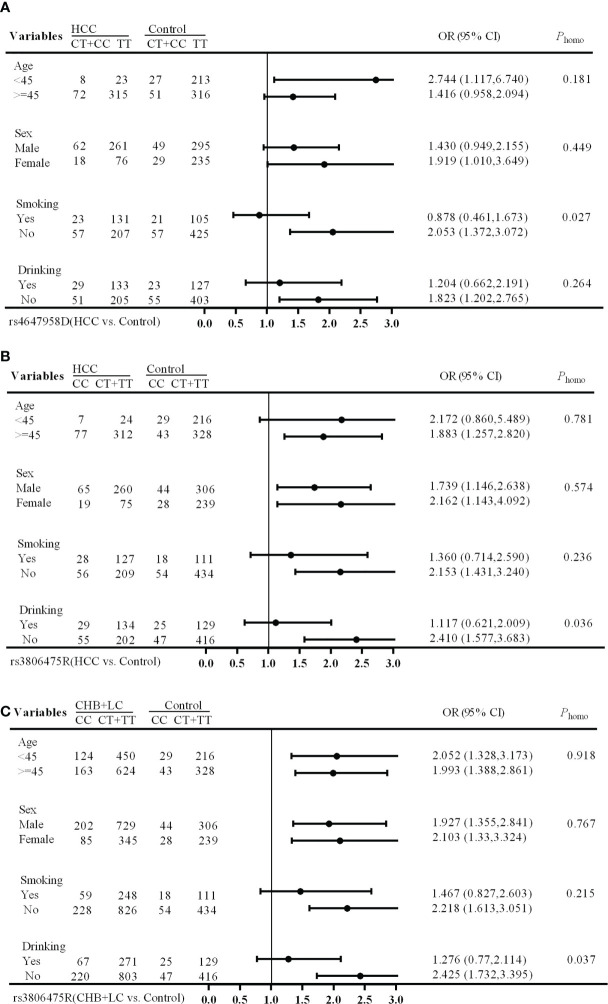
Stratification analysis of associations between EMT regulatory genes and HBV-related HCC risk. **(A)** HCC *vs* Health Control (rs4647958D); **(B)** CHB+LC *vs*. Health Control (rs3806475R); **(C)**, HCC *vs* Health Control (rs3806475R). CHB, chronic hepatitis B; LC, liver cirrhosis; HCC, hepatocellular carcinoma; Phomo from the homogeneity test in each stratum was tested by the Breslow-Day Test.

The stratification analysis showed that the rs4647958 genotype-associated risk of HCC development was more pronounced in non-smoking individuals (*OR*, 2.053; 95% *CI*, 1.372-3.072) versus those who did smoke (*OR*, 0.878; 95% *CI*, 0.461-1.673; Breslow-day test, *P* = 0.027) under the dominant model (see **Figure 1A**). Meanwhile, the rs3806475 genotype-associated risk of HCC development was more pronounced in non-drinking individuals (*OR*, 2.410; 95% *CI*, 1.577-3.683) versus those who did drink (*OR*, 1.117; 95% *CI*, 0.621-2.009; Breslow-day test, *P* = 0.036) under the recessive model (see **Figure 1B**). Last, the rs3806475 genotype-associated risk of CHB+LC development was more pronounced in non-drinking individuals (*OR*, 2.425; 95% *CI*, 1.732-3.395) compared with those who did drink (*OR*, 1.276; 95% *CI*, 0.770-2.114; Breslow-day test, *P* = 0.037) under the recessive model (see **Figure 1C**). None of the other SNPs observed were associated with any significant differences in disease characteristics.

### *SNAIL1*, *ZEB2* and *TWIST1* Genotypes and Their Correlation With HbsAg-Positive HBV With and Without HCC Progression

EMT has been widely studied in the metastatic process of epithelial malignancies (18). We therefore analysed the correlation between SNPs and HCC clinical stages as shown in **Supplementary Table 3**. We found that the rs4647958 *SNAIL1* genotypes were correlated with HCC progression with a lower correlation-coefficient in non-smoking patients (*r*_s_= 0.087, *P*<0.001). Additional correlations were identified in the following patient groups: age less than 45 years (rs4647958: *r*_s_= 0.113, *P*=0.001), female (rs4647958: *r*_s_= 0.079, *P*=0.026; rs2285681: *r*_s_= 0.074, *P*=0.038) and non-drinking (rs4647958: *r*_s_= 0.074, *P*=0.002; rs3806475: *r*_s_= 0.054, *P*=0.025).

### LD and Haplotype Block Analysis

Haplotype block LD mapping demonstrated that the rs2285681 and rs2285682 SNPs are in tight LD in a 0-kb sequence, while the rs4647958 and rs1543442 SNPs are in tight LD in a 4-kb sequence (**Supplementary Figure 1**). As shown in **Table 3**, the CG haplotype of the rs4647958/rs1543442 haplotype block is associated with significant differences between healthy control subjects and HCC patients (*P*=0.0347). Meanwhile, the CT haplotype of the rs2285681/rs2285682 haplotype block is associated with significant differences between CHB+LC and HCC patients (*P*=0.0123). However, no significant correlations were identified between other observed SNPs.

**Table 3:** Haplotype analysis between HCC and patients with CHB + LC by Haploview.

	Haplotype	Freq.	Case, Control Ratio Counts	Case, Control Frequencies	Chi Square value	*P* value
Comparison between health control and HCC						
Block 1	GT	0.729	598.4: 243.6, 912.9: 319.1	0.711, 0.741	2.327	0.1271
	CT	0.157	147.6: 694.4, 178.8: 1053.2	0.175, 0.145	3.431	0.0640
	CG	0.114	96.0: 746.0, 140.3: 1091.7	0.114, 0.114	<0.001	0.9906
Block 2	TG	0.536	450.8: 389.2, 660.6: 573.4	0.537, 0.535	0.003	0.9535
	TA	0.382	307.7: 532.3, 485.5: 748.5	0.366, 0.393	1.562	0.2114
	CG	0.082	81.5: 758.5, 87.9: 1146.1	0.097, 0.071	4.458	0.0347
Comparison between CHB+LC and HCC						
Block 1	GT	0.722	598.4: 243.6, 1985.4: 750.6	0.711, 0.726	0.723	0.3952
	CT	0.149	147.6: 694.4, 383.7: 2352.3	0.175, 0.140	6.266	0.0123
	CG	0.128	95.9: 746.1, 363.4: 2372.6	0.114, 0.133	2.066	0.1506
Comparisons between health control and CHB+LC						
Block 1	GT	0.730	1985.7: 750.3, 912.8: 321.2	0.726, 0.740	0.842	0.3589
	CT	0.142	383.4: 2352.6, 178.7: 1055.3	0.140, 0.145	0.156	0.6931
	CG	0.127	363.4: 2372.6, 141.2: 1092.8	0.133, 0.114	2.579	0.1083

Block 1, rs2285681 and rs2285682; Block 2, rs4647958 and rs1543442.

### MDR Models of Environment-Gene and Gene-Gene Interactions

**Figure 2:**
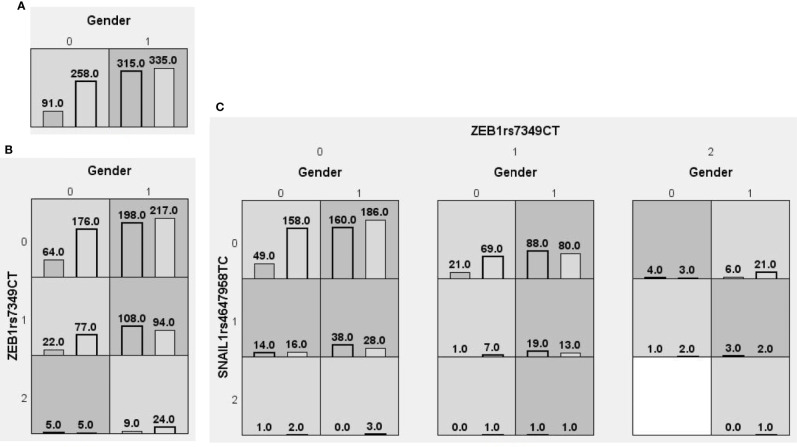
Distributions of high-risk and low-risk genotypes between HCC and health control. **(A)** Single-locus model; **(B)** Two-locus model; **(C)** Three-locus model. Dark gray and light gray boxes presented the high-risk and low-risk SNP combinations, respectively. Left bars inside each box represented major depressive disorder while the right bars represented control. The heights of the bars are proportionate to the sum of samples in each group. The patterns of high-risk and low-risk cells differ across each of the different multi-locus SNP dimensions.

**Figure 3:**
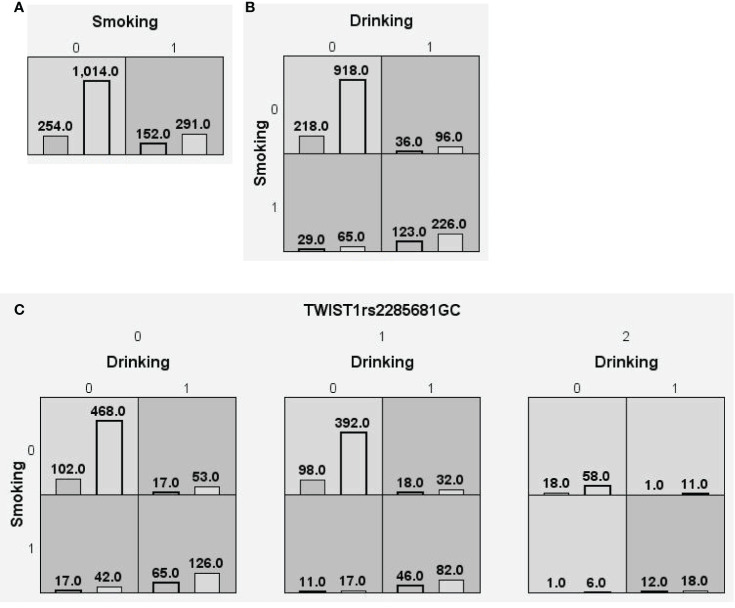
Distributions of high-risk and low-risk genotypes in between HCC and CHB+LC patients. **(A)** Single-locus model; **(B)** Two-locus model; **(C)** Three-locus model. Dark gray and light gray boxes presented the high-risk and low-risk SNP combinations, respectively. Left bars inside each box represented major depressive disorder while the right bars represented control. The heights of the bars are proportionate to the sum of samples in each group. The patterns of high-risk and low-risk cells differ across each of the different multi-locus SNP dimensions.

**Figure 4:**
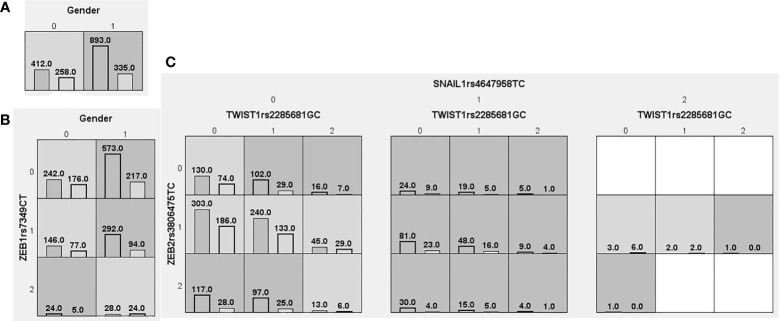
Distributions of high-risk and low-risk genotypes in between CHB+LC patients and health control. **(A)** Single-locus model; **(B)** Two-locus model; **(C)** Three-locus model. Dark gray and light gray boxes presented the high-risk and low-risk SNP combinations, respectively. Left bars inside each box represented major depressive disorder while the right bars represented control. The heights of the bars are proportionate to the sum of samples in each group. The patterns of high-risk and low-risk cells differ across each of the different multi-locus SNP dimensions.

We searched for possible genetic interactions of the four genes studied in the context of HCC. We evaluated up to three-locus interactions with 6 polymorphic sites and 3 environmental factors (gender, tobacco smoking and alcohol drinking). For HCC and health subjects as comparative groups, gender in one-locus models was the best, while the balanced accuracy (BA) for testing the dataset was 60.55% and the CVC was 10/10. For HCC and CHB+LC as comparative groups, the combination drinking, smoking in the two-locus model was the best, while the BA was 57.48% and the CVC was 9/10. For CHB+LC and health subjects as comparative groups, the combination *TWIST1*rs2285681*, ZEB2*rs3806475*, SNAIL1*rs4647958 of the three-locus model was the best model with a BA of 56.99% and CVC of 9/10. **Table 4** summarizes the MDR results for the one- to three-locus models. **Figures 2**–**4** show the detailed distribution of high- and low-risk genotypes in the best three-locus model for the HCC and CHB+LC. These results were all significant, with empirical *p*-values of <0.001 in 10000 permutation tests.

**Table 4:** MDR models of analyse the environment-gene and gene-gene interactions.

Comparative group	Model	Training Balanced Accuracy (%)	Testing Balanced Accuracy (%)	Cross Validation Consistency	Chi Square value	*p*-value
HCC *vs* Health	Gender	60.55	60.55	10/10	47.1755	<0.0001
	Gender, *ZEB1* rs7349	61.69	60.32	8/10	56.0426	<0.0001
	Gender, *SNAIL1* rs4647958, *ZEB1* rs7349	62.76	58.77	5/10	67.1358	<0.0001
HCC *vs* CHB+LC	Smoking	57.63	56.09	7/10	36.9906	<0.0001
	Drinking, Smoking	58.34	57.48	9/10	38.4753	<0.0001
	Drinking, Smoking, *TWIST1* rs2285681	58.99	55.13	6/10	43.0362	<0.0001
CHB+LC *vs* Health	Gender	56.00	54.91	8/10	25.4365	<0.0001
	Gender, *ZEB1* rs7349	57.51	55.74	6/10	38.7054	<0.0001
	*TWIST1* rs2285681, *ZEB2* rs3806475, *SNAIL1* rs4647958	58.67	56.99	9/10	50.6300	<0.0001

## Discussion

We hypothesized that EMT genes play an important role in HCC and chronic HBV infection, and that environment-gene and gene-gene interactions are important. We found significant genetic associations for single EMT genes with HCC and chronic HBV infection, as well as environment-gene and gene-gene interactions. The MDR results indicated that interactions of environment-gene and gene-gene contribute significantly to HCC and chronic HBV infection, even when individual EMT genes do not.

In this study, we found that EMT-related genes were important in HCC and chronic HBV infection. Our findings demonstrate that the *SNAIL1* rs4647958T>C, *ZEB2* rs3806475T>C and *TWIST1* rs2285681G>C SNPs are associated with increased susceptibility to both HCC and chronic HBV infection. In addition, interactions among potentially related polymorphic sites were associated with the development of HCC through the MDR method. MDR is a suitable method to analyse environment-gene and gene-gene interactions by reducing multi-locus genotypes into high-risk and low-risk groups in case-control studies (19). This method marks the genotype in each cell as high or low risk based on whether the ratio of case to control cell is greater than or less than the threshold (20). Furthermore, the MDR method is further extended to model-based MDR, generalized MDR and surviving MDR, etc., to apply to different situations (21–23). Our study found *SNAIL1* rs4647958 showed a significant environment-gene interaction for chronic HBV infection with or without HCC in the MDR results. *TWIST1* rs2285681 showed a significant environment-gene interaction for the development of HCC.

For HCC and health subjects as comparative groups, the one-locus model was found to be optimal for the prediction of HCC in terms of BA (60.55%). However, the BA values between the two- and three-locus combination models in HCC didn’t have any meaningful difference. For HCC and CHB+LC as comparative groups, the two-locus model was found to be the best in terms of BA (57.48%). Similarly, the BA values between the one- and three-locus combination models didn’t have any meaningful difference. For CHB+LC and health subjects as comparative groups, the four-locus combination model (*TWIST1* rs2285681, *ZEB2* rs3806475 and *SNAIL1* rs4647958) was the best for BA (56.99%) and the CVC was 9/10.

In the three-locus combination models for HCC and CHB+LC, *SNAIL1* rs4647958 was common to both liver diseases, while the other two factors differed. Comparatively, while we compared HCC and CHB+LC, CHB+LC and health subjects, *TWIST1* rs2285681 appeared in both three-locus combination models at the same time. Our finding of the EMT-related gene interaction seemed to support the clinical observation that *SNAIL1* rs4647958 and *TWIST1* rs2285681 had an impact on patients with HCC and chronic HBV.

In this study, we found significant associations between germline variants of six EMT regulators and the development of chronic HBV infection and HCC revealed that the *SNAIL1* exon variant rs4647958T>C and the *ZEB2* promoter exon variant rs3806475T>C are significantly associated with the risk of developing both diseases. Additionally, the *TWIST1* promoter exon variant rs2285681G>C is associated with an increased risk of HBV-related HCC. Furthermore, the *SNAIL1* rs4647958T>C genotype is associated with decreased probability of HBV-related HCC metastasis at diagnosis among smokers.

The stratified analysis showed that *SNAIL1* genotypes (rs4647958) are associated with the development of a more aggressive form of HCC in non-smokers, *ZEB2* genotypes (rs3806475) are associated with increased risk of HCC development in non-drinkers, and *TWIST1* genotypes (rs3806475) are associated with increased risk of CHB and LC. Meanwhile, the *SNAIL1* SNPs (rs4647958) are correlated with HCC stages in smokers, though not significantly. *SNAIL1* is an important factor involved in inducing and promoting EMT. *SNAIL1* is also involved in the pathogenesis of hepatitis B virus mutations in HCC patients. Our findings are remarkably consistent with previously published studies. Chen et al. (24) found that *SNAIL* is negatively correlated with E-cadherin expression and positively correlated with *MMP-2* expression in HCC tissues. Further, these changes in E-cadherin and MMP-2 expression help to promote HCC invasion. Woo et al. (25) used immunohistochemistry to study HCC and found that *SNAIL* expression is correlated with low E-cadherin expression and poor differentiation in hepatocellular carcinoma. The occurrence and development of HCC are related to many signal pathways, and the expression of *SNAIL* can play a role in the process of HCC by affecting these signal pathways. Kim et al. (26) found that Notch1 and ROS synergistically upregulate the expression of *SNAIL* protein in hepatoma carcinoma cells through the PI3K/Akt signalling pathway, thereby increasing cancer cell invasion. Cheng et al. (27) demonstrated that increased expression of *SNAIL1* can promote liver tumour initiation, progression, and metastasis. High *SNAIL1* expression was also reported in liver tissues, suggesting that it also contributes to HCC pathogenesis (28). These pieces of evidence all indicate that the *SNAIL* variant (rs4647958) is functional and contributes to increased risk of HCC and chronic HBV infection.

The *TWIST1* protein (also known as *Twist*) can regulate the expression of many specific genes and participates in many different biological processes required for normal growth and development (29). However, *TWIST* also plays an oncogenic role in tumour cells. Yang et al. (30) showed that *TWIST* plays a key role in the vascular invasion and lung metastasis of cancer cells. During the process of tumour metastasis, primary tumour cells undergo EMT and then metastasize to distant organs *via* the circulatory system. *TWIST* stimulates tumour metastasis by promoting the occurrence of EMT in tumour cells. In addition, *TWIST* can inhibit apoptosis and senescence pathways and immortalize cells (31).

The *ZEB2* protein plays an important role as a transcription factor in the *TGF* signalling pathway. This signalling pathway is essential during early foetal development (32). *ZEB1* and *ZEB2* can bind to the CACCT (G) sequence in the promoter of the E-cadherin gene, causing epithelial cells to lose their epithelial-like characteristics and transform into mesenchymal cells, thus leading to EMT (33). Gene mutations can result in the production of non-functional *ZEB2* proteins or can completely inactivate the gene. The absence of *ZEB*2 proteins influences the biological processes of many organs. *ZEB2* mutations are the underlying cause of irregular development of the neural crest (34). Our study is the first to demonstrate that mutations in the *ZEB2* gene are related to HCC.

As a case-control hospital-based study, some limitations in our study are inevitable. For example, selection and information biases are unavoidable. However, our identification of associations between gene variants and HBV-related HCC risk are unlikely to be solely due to chance, as these findings were confirmed by the results of functional assays.

Longo et al. (35) concluded that the liver microenvironment of HCC patients is more immunosuppressed, accompanied by an increase in the number of regulatory T cells (Tregs), tumor-associated macrophages (TAM) and myeloid-derived suppressor cells (MDSC), which is associated with tumor progression and poor prognosis. Here we described and analysed the single-gene associations and environment-gene/gene-gene interactions of EMT-related genes with HBV-related HCC. We found that EMT genes play a role in HBV-related HCC and genetic factors at multi-levels, from alleles and genotypes to haplotypes and environment-gene/gene-gene interactions. Our study suggests that these SNPs are not only candidate predictors for HCC and chronic HBV infection risk but may also be a genetic determinant for the development of HCC in the chronic HBV infection population. Future studies should be performed in a larger population encompassing multiple ethnic groups in order to confirm our findings.

## Data Availability Statement

The datasets presented in this study can be found in online repositories. The names of the repository/repositories and accession number(s) can be found in the article/**Supplementary Material**.

## Ethics Statement

The studies involving human participants were reviewed and approved by Ethics Committee of Hebei Medical University. Written informed consent to participate in this study was provided by the participants’ legal guardian/next of kin. Written informed consent was obtained from the individual(s), and minor(s)’ legal guardian/next of kin, for the publication of any potentially identifiable images or data included in this article.

## Author Contributions

Conceptualization: W-XL, LY, XG, and D-WL. Data curation: L-NY, NM, X-LZ, and L-MT. Data analysis: XG, W-XL, LY, and D-WL. Funding acquisition: XG and D-WL. Investigation: LY, H-MY, L-NY, NM, X-LZ, L-MT, and XG. Methodology: XG, D-WL, and L-MT. Project administration: D-WL, XG, and LY. Resources: XG and D-WL. Supervision: XG, D-WL, W-XL, and LY. Writing the original draft: XG and D-WL. Reviewing and editing: W-XL, LY, H-MY, L-NY, NM, X-LZ, L-MT, XG, and D-WL. All authors contributed to the article and approved the submitted version.

## Funding

This study received financial support from Department of Education of Hebei Province (grant number QN2017101,BJ2019019), Science and Technology Bureau of Hebei Province (grant number 17272407), National Natural Science Foundation of China (grant number 81601876), and National Natural Science Foundation of Hebei Province (grant number H2019206528).

## Conflict of Interest

The authors declare that the research was conducted in the absence of any commercial or financial relationships that could be construed as a potential conflict of interest.

## Abbreviations

HCC, hepatocellular carcinoma; HBV, hepatitis B virus; CHB, chronic hepatitis B; LC, liver cirrhosis; EMT, epithelial-mesenchymal transition; SNP, single-nucleotide polymorphism; LD, linkage disequilibrium; MDR, multifactor dimensionality reduction; MAF, minor allele frequency; mean ± SD, mean ± standard deviation; M(QR), median(quartile interval); OR, odds ratio; CI, confidence interval; EDTA, ethylenediamine-tetra-acetic acid; 3'-UTR, 3'-untranslatedregion.
