# Supplementary material for: Germline Variants and Genetic Interactions of Several EMT Regulatory Genes Increase the Risk of HBV-Related Hepatocellular Carcinoma
Source: Front Oncol. 2021 Jun 11;11:564477. doi: 10.3389/fonc.2021.564477 (PMC8226114; doi:10.3389/fonc.2021.564477)

Supplemental Methods

1. Linkage disequilibrium (LD) and haplotype block analyses

In a certain population, there is a significant difference between the frequency of two alleles at different loci in the same haplotype and the expected random frequency, which is called linkage disequilibrium (linkage disequilibrium). Such non-random combinations of certain alleles of different loci are often inherited together. LD analysis is to investigate the linkage disequilibrium between loci through D’/r^2 and so on.

The alleles of adjacent SNPs tend to be passed on to offspring as a whole. A group of related SNP alleles located in a certain region on the chromosome is called haplotype. Haplotype analysis uses Pearson's chi-square test to examine whether these overall inherited haplotypes are related to disease.

2. Multifactor dimensionality reduction (MDR) method

MDR is a multi-factor dimensionality reduction method. In this method, “factor” is a variable in the interaction study (such as genotype or environmental factors), and “dimension” refers to the number of factors (such as genotype) in the multi-factor combination studied, classified by disease susceptibility (high risk or low-risk) method to establish a model, the multiple factors in the study as a multi-factor combination (genotype combination), so that the high-dimensional structure is reduced to two levels (high-risk or low-risk) of one-dimensional, namely for "dimensionality reduction".

When using MDR to analyze interaction effects, it is generally required that the response variable be a binary variable, such as a case or a control (valid or invalid outcome, etc.), and the predictor variable shall be a mutually independent categorical variable, such as environmental factors or genotyping. Through the largest cross-validation consistency (CVC) and the best balanced accuracy (BA), the best n-factor interaction model for predicting the risk of HCC is determined. Draw the best model distribution diagram and interactive tree diagram to explain the interaction of various factors in the best prediction model.

The analysis steps of MDR are as follows.

Step 1 This research adopts a ten-fold crossover study, and randomly divides the sample into 10 parts, 9 of which are training samples and 1 is a test sample.

Step 2 Select n factors from many research factors to form different combinations of n factors (for example, 3 genes, three genotypes for each gene, there are 27 combinations), these factors can be SNPs or clearly classified environments factor.

Step 3 According to the different levels of n factors, individuals are divided into different categories, as shown in the cells in the figure, the left band represents the case, and the right band represents the control.

Step 4 Calculate the ratio of the number of cases to the number of controls in each grid. If the ratio of cases to controls is greater than a certain threshold (for example, ≥1), it is marked as high risk, otherwise, it is marked as low risk. So that the high-dimensional structure is reduced to two levels (high-risk or low-risk) of one-dimensional.

Step 5 The set of multi-factor classification contains the combination of the factors in the MDR model. Among all the combinations, the MDR model with the smallest individual misclassification is selected, which has the smallest prediction error among all models.

Step 6 The prediction error of the model is evaluated through ten-fold cross-validation, the model with the smallest prediction error is selected as the final model, and the average of the prediction errors of 10 tests is taken as an unbiased estimate of the model-related prediction errors.

The steps of MDR method are shown in the chart below.


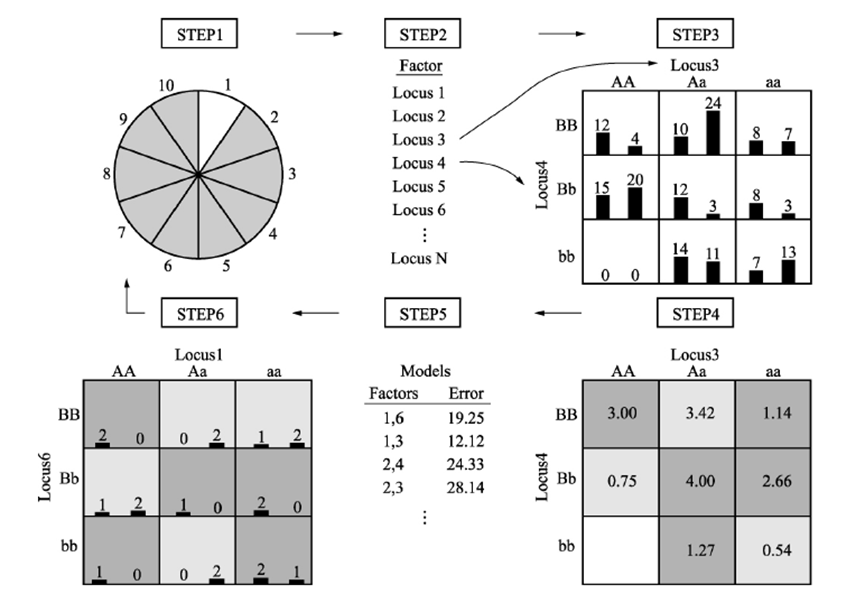


Supplementary Table 1 Information of primer sequences for MassARRAY allelic discrimination

| Polymorphism | Sequence | |
| --- | --- | --- |
| rs4647958T>C | 1st-PCRP | ACGTTGGATGTCCTTCGTCCTTCTCCTCTA |
|  | 2nd-PCRP | ACGTTGGATGAAGCCTGGGAAGGCAGCATA |
|  | UEP_SEQ | GGAGCGGCCTCCAAGGAAGAG |
| rs1543442G>A | 1st-PCRP | ACGTTGGATGTGCCCTCCCTCCACAGAAA |
|  | 2nd-PCRP | ACGTTGGATGTTCTGGTTCTGTGTCCTCTG |
|  | UEP_SEQ | GGAAGAGGCCTTCCC |
| rs7349C>T | 1st-PCRP | ACGTTGGATGCCTAATTTGGCTTACTAAGG |
|  | 2nd-PCRP | ACGTTGGATGATAGCATGATGCTCTGCAGT |
|  | UEP_SEQ | TGCTCTGCAGTTTTATTAAGAAAT |
| rs3806475T>C | 1st-PCRP | ACGTTGGATGAGGCATTCAGGATCTTCAGC |
|  | 2nd-PCRP | ACGTTGGATGAAAATTAAGAATGTGCCTG |
|  | UEP_SEQ | TGTGCCTGACCCATG |
| rs2285681G>C | 1st-PCRP | ACGTTGGATGTTAATTGAGCAAAGCGCCCC |
|  | 2nd-PCRP | ACGTTGGATGAGCTTGAGATATCTGCAGCC |
|  | UEP_SEQ | GGGGCTATCTGCAGCCGCGAACCTTG |
| rs2285682T>G | 1st-PCRP | ACGTTGGATGATGGGAGGGACAAATTGCAG |
|  | 2nd-PCRP | ACGTTGGATGAAACAGGCCAGTTGACAAGC |
|  | UEP_SEQ | AGTAACTTTCTGAGTATTTCTGTAAAA |

Supplementary Table 2 The demographic parameters of study subjects

| Variable | Health Control  (*N*=618) | CHB+LC  (*N*=1371) | HCC  (*N*=421) | Chi square value | *P*-value |
| --- | --- | --- | --- | --- | --- |
| Age(years, *M*(*QR*) | 49 (22) | 47 (21) | 57 (12) | 216.293 | **<0.001**^a^ |
| Gender(Male/Female) | 350/268 | 936/435 | 326/94 | 52.380 | **<0.001** ^b^ |
| Tobacco Smoking(Yes/No) | 129/489 | 307/1064 | 156/265 | 43.477 | **<0.001** ^c^ |
| Alcohol drinking(Yes/No) | 154/464 | 339/1032 | 164/257 | 35.183 | **<0.001** ^d^ |

The bonfferny method was used for pairwise comparisons between groups when there was a significant difference in the overall distribution of each factor in the three groups.

^a^ The HCC group had significantly higher mean age than the other two groups, and health control group had significantly higher mean age than CHB+LC group .

^b^ The HCC group had significantly higher ratio of males than the other two groups, and CHB+LC group had significantly higher ratio of males than health control group.

^c^ The HCC group had significantly higher ratio of smokers than the other two groups, but there was no statistical significance between health control and CHB+LC groups.

^d^ The HCC group had significantly higher ratio of drinkers than the other two groups, but there was no statistical significance between health control and CHB+LC groups.

Supplementary Table 3 The correlation between the SNPs of EMT and clinical stages of HCC tested by Spearman’s Rank Correlation

|  | rs4647958 | | rs3806475 | | rs2285681 |  |
| --- | --- | --- | --- | --- | --- | --- |
|  | *r*_s_ | *P* | *r*_s_ | *P* | *r*_s_ | *P* |
| Total | 0.059 | **0.004** | 0.015 | 0.455 | 0.033 | 0.107 |
| Age  <45 | 0.113 | **0.001** | 0.004 | 0.225 | 0.020 | 0.556 |
| ≥45 | 0.042 | 0.103 | 0.004 | 0.874 | 0.037 | 0.145 |
| Gender |  |  |  |  |  |  |
| Male | 0.040 | 0.108 | 0.017 | 0.508 | 0.018 | 0.486 |
| Female | 0.079 | **0.026** | 0.009 | 0.791 | 0.074 | **0.038** |
| Smoking status  Yes | -0.020 | 0.634 | -0.056 | 0.173 | 0.053 | 0.202 |
| No | 0.087 | **<0.001** | 0.041 | 0.081 | 0.032 | 0.170 |
| Drinking status  Yes | 0.021 | 0.591 | -0.084 | **0.031** | 0.022 | 0.577 |
| No | 0.074 | **0.002** | 0.054 | **0.025** | 0.040 | 0.096 |

Supplementary Figure 1. Block for HBV-Related HCC of EMT regulatory genes single-nucleotide polymorphisms. A, Comparison between health control subjects and HCC patients; B, Comparison between CHB+LC patients and HCC patients; C, health control subjects and CHB+LC patients. CHB, chronic hepatitis B; LC, liver cirrhosis; HCC, hepatocellular carcinoma.


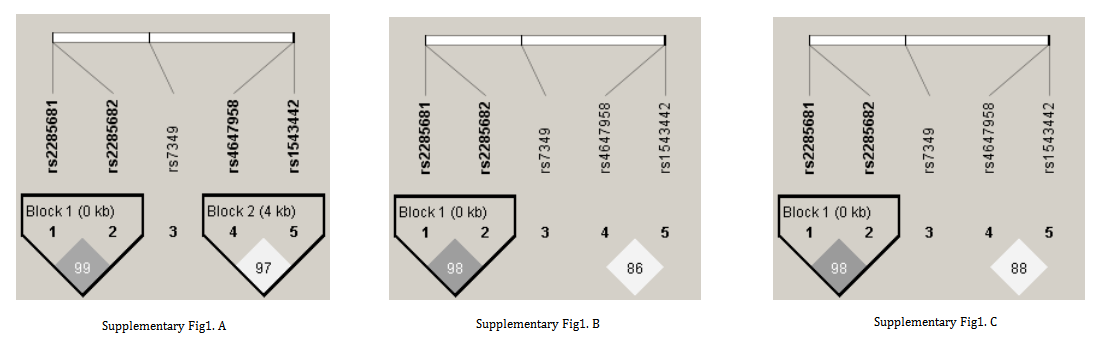

Supplement: Supplementary file 1 [file DataSheet_1.docx]
